# Continuous subcutaneous insulin infusion is associated with a better glycemic control than multiple daily insulin injections without difference in diabetic ketoacidosis and hypoglycemia admissions among Emiratis with Type 1 diabetes

**DOI:** 10.1371/journal.pone.0264545

**Published:** 2022-09-22

**Authors:** Raya Almazrouei, Charu Sharma, Bachar Afandi, Khaled M. Aldahmani, Elhadi H. Aburawi, Salem A. Beshyah, Gehad ElGhazali, Zain Al Yafei, Rami H. Al-Rifai, Juma Alkaabi

**Affiliations:** 1 Division of Endocrinology, Tawam Hospital, Al Ain, United Arab Emirates; 2 Department of Internal Medicine, College of Medicine and Health Sciences, Al Ain, United Arab Emirates University, United Arab Emirates; 3 Department of Pediatrics, College of Medicine and Health Sciences, United Arab Emirates University, Al Ain, United Arab Emirates; 4 Department of Medicine, Dubai Medical College, Dubai, United Arab Emirates; 5 Department of Immunology, Sheikh Khalifa Medical City and Union71/Purehealth, Faculty of Medicine, United Arab Emirates University, Abu Dhabi, United Arab Emirates; 6 Union71, Purehealth, Abu Dhabi, United Arab Emirates; 7 Institute of Public Health, College of Medicine and Health Sciences, United Arab Emirates University, Al Ain, United Arab Emirates; Sohag University Faculty of Medicine, EGYPT

## Abstract

**Aims:**

To characterizes Emiratis patients with Type 1 diabetes (T1D) and compares outcomes between continuous subcutaneous insulin infusion (CSII) versus multiple daily insulin injections (MDI) users. The WHO-Five Well-Being Index (WHO-*5*) score was used to screen for depression.

**Methods:**

In this cross-sectional study; sociodemographic, clinical characteristics and insulin replacement regimens were collected on patients with T1D between 2015–2018.

**Results:**

134 patients with mean age of 20.9±7.5 years were included. Females constitute 56.7% and 50.7% had diabetes duration of >10 years. Diabetic ketoacidosis (DKA) at presentation was reported in 46.3%. Average glycemic control over preceding 12months was satisfactory (less than 7.5%), suboptimal (7.5–9%), and poor (more than 9%) in 26.6%, 42.7% & 30.6% of the patients, respectively. Higher proportion of patients using CSII achieved satisfactory or suboptimal glycemic control compared to patients with MDI (P = 0.003). The latest median /IQR HbA1c was significantly lower (P = 0.041) in patients using CSII (8.2 /1.93%) compared to MDI (8.5/2.45%). There was no significant difference between two groups in DKA, severe hypoglycemia or total WHO-*5* score.

**Conclusions:**

CSII usage was associated with better glycemic control than MDI, although no difference in DKA and severe hypoglycemia. The overall glycemic control among Emiratis subjects with T1D is unsatisfactory and needs more rigorous patient counseling and education.

## 1. Introduction

Type 1 Diabetes (T1D) is a globally rising autoimmune disease involving the dysfunction of pancreatic β-cells by the immune system, thus diminishing insulin production. The International Diabetes Federation (IDF) [1] estimates that more than a million individuals (age <20 years) live with T1D worldwide. Although the Arab populations of the Middle East represent only 5.4% of the total world population, they contribute remarkably to the increasing global burden of T1D, with 60000 cases reported in children with age <14 years [2]. Two countries from the Arab region are among the top 10 countries globally with the highest incidence rate of T1D are Kuwait (44.5/100000) and Saudi Arabia (33.5/100000) [1,3,4]. Limited data have been published on the incidence of T1D in the United Arab Emirates [5]. On the other hand, there continues to be uncertainty about the best insulin replacement regime in T1D. The comparative effectiveness of continuous subcutaneous insulin infusion (CSII) versus multiple daily insulin injections (MDI) showed mixed results with variable reported effectiveness of CSII over MDI [6]. Older studies reported different results with either no difference between the two treatment modalities in terms of glycemic control and risks of severe hypoglycemia and DKA [7] or superiority of CSII therapy in patients with T1D [8]. However, more recent data showed advantages of CSII over MDI in glycemic control and severe hypoglycemia and DKA [9].

This cross-sectional study describes Emirates patients’ sociodemographic and clinical characteristics with T1D and investigates the impact of CSII and MDI usage on several clinical outcomes, particularly glycemic control. We also assess the World Health Organization-Five Well-Being Index (WHO-5) as a screening method for depression in this cohort of T1D patients.

## 2 Patients and methods

### 2.1 Study population

In this cross-sectional study, a total of 134 patients with T1D, under regular follow-up at two specialized T1D clinics at Sheikh Khalifa Medical City in Abu Dhabi and Tawam Hospital in Al-Ain, were recruited. Patients were conveniently selected to be included in this study as long as they are diagnosed with T1D. The diagnosis of T1D was based on the 1985 World Health Organization (WHO) diagnostic criteria (WHO Technical Report Series 727; Geneva 1985). Patients with T1D were included if they were diagnosed with T1D on a clinical and laboratory basis (C-peptide levels < 0.3 mmol/l) and managed with insulin therapy since they were diagnosed. Patients diagnosed with other types of diabetes were excluded, including maturity-onset diabetes of the young (MODY), type 2 diabetes, and polyendocrinopathies.

### 2.2 Data collection and definitions of variables

Information from medical charts and face-to-face interviews were collected included age, gender, family size, patient’s and parent’s educational attainment, occupation, smoking status, comorbidities, and family history of T1D or autoimmune diseases. Family support was assessed subjectively by the interviewer by asking the following kinds of questions without specific scoring: Do they understand your condition? Do they help you to take the medication or remind you? Do they know type of insulin you are taking? Do they know how to act if you develop hypoglycemia? Do they help and support with the type of food and meals to be taken?

Information on the duration of the T1D, mode of T1D presentation, insulin administration regime (CSII or MDI), body mass index (BMI), blood pressure, lab tests, daily frequency of blood glucose monitoring, and continuous glucose monitoring (CGM) or flash glucose monitoring (FGM) use were collected. For the sake of comparison between treatment regimes, all included patients has been on their treatment regimen for at least the preceding 12 months. During the study period, the licensed insulin pumps were MiniMed paradigm from Medtronic and Accu-Chek from Roche. The licensed CGM and FGM included Enlite glucose sensor from Medtronic and Freestyle libre from Abbott. The average HbA1c over the previous 12 months, as indicative of glycemic control, defined as poor (more than 9%), suboptimal (7.5–9%), and satisfactory (less than 7.5%) [10] was recorded. In these two specialized T1D clinics, patients are reviewed every 3 months with three monthly HbA1c. Keeping in mind, that some patients will have less than four HbA1c readings per year, we calculated average HbA1c when at least 2 readings are available over the year. We did not collect how many readings have been counted in each patient. The frequency of physician, dietician, and diabetic educators’ visits was also counted. All patients with T1D had education and training on carbs counting and how to use insulin sensitivity factors for calculating correction boluses irrespective of treatment regimen. Severe Hypoglycemia in this study was defined as hypoglycemia requiring third part assistance from either surroundings or rapid response emergency team or presentation to the hospital. It was collected from direct interview with the patients as such severe hypoglycemic episodes are usually remembered by most of the patients. The frequency of DKA episodes was determined from face-to-face interview and confirmed with chart review. Autoimmune diseases reported by the patients were confirmed with chart review. Thyroid disorders were defined as either diagnosis with hypothyroidism or harboring thyroid peroxidase antibodies. Most patients will have annual thyroid function test, while thyroid peroxidase antibodies were performed based on physician discrete decision usually in case of elevated TSH. Celiac disease and adrenal disease were defined as confirmed disease with the appropriate confirmatory testing in each. Celiac disease screening was variable based on physician practice whether annual screen is done with tissue transglutaminase antibodies or screening only symptomatic patients. Adrenal disease only in patient who is symptomatic, and no regular screening performed.

World Health Organization-Five Well-Being Index (WHO-5) as a screening method for depression was used in this cohort of T1D patients. WHO-5 is a short questionnaire to assess the level of well-being over 14 days. It consists of five simple questions, which tap into the subjective well-being of the respondents. The raw score ranges from 0 (absence of well-being) to 25 (maximal well-being) that then multiply by 4 to translate to a percentage scale from 0 (absent) to 100 (maximal) [11]. WHO-5 index appears suitable for use as a screening test for likely depression in outpatients with Type 1 and Type 2 diabetes with different proposed raw score cutoff ranging from 7 to 13 [12,13].

### 2.3 Statistical analysis

Descriptive and correlation analyses were performed. In descriptive analysis, we described patients with T1D according to their measured sociodemographic and clinical characteristics. Frequencies and proportions of categorical variables were quantified and presented. To assess the difference in the proportion of patients with T1D according to the two treatment modalities (CSII and MDI) and measured categorical sociodemographic (e.g. gender, education) and clinical characteristics, Chi-square test was used.

The normality assumption of the continuous variables was tested using the Shapiro-Wilk test. Means and standard deviations (SD) of normal distributed continuous variables while medians and interquartile ranges (IQR) of the non-normal distributed continuous variables were quantified and presented. The independent two-sample t-test was used to compare means and SD of normal-distributed continuous variables (e.g. age and BMI), while the Mann-Whitney U test was used to assess the statistical difference between the non-normal distributed variables. The p-value from both tests was obtained and reported

Statistical significance difference between T1D using and measured variables was set at a p-value < 0.05. All data analyses were preformed using the SPSS Statistics v25 software (I.B.M. Corp., Armonk, NY, U.S.A.).

### 2.4 Ethical approval

This study was conducted in accordance with the Declaration of Helsinki after the institutional ethical committee approval from Sheikh Khalifa Medical City [REC-25.10.2016 (RS-445)] and Al Ain Medical District Human Research Ethics committee (AAMDHREC) (ERH-2016-4255 16–002). Written informed consent was obtained from all the recruited subjects and the parents or guardians of minors involved in this study.

## 3. Results

The study included 134 patients with T1D from the two study centers. Table 1 shows the sociodemographic characteristics in comparison between T1D patients based on insulin administration regimen (CSII vs. MDI). Almost half of the patients (49.3%) were using CSII therapy while the other half on multiple (> = 4) daily insulin injections. The mean age of the patients was 20.96 ± 7.45 years; among them, 56.7% were females, and 43.3% were males. More female patients tend to use CSII therapy (66.7%) than males (33.3%). Almost half of the patients (46.3%) presented with diabetes ketoacidosis (DKA) at the time of diagnosis. An equivalent number was diagnosed with symptomatic hyperglycemia without DKA. Half of the patients had diabetes for more than ten years, while 27.6% had diabetes between 5–10 years, and 21.6% had diabetes of less than five years. There was no difference in T1D duration between CSII and MDI users. However, among the CSII group, the proportion of users was greater with a longer disease duration. A statistically significantly higher proportion of patients with education level of high school and above were using CSII therapy (85.7%) compared to MDI (68.7%). There was no significant difference between the two insulin regimens based upon the mother’s education, family size, and adequate support in the present study. Almost 80% of the patients reported a family size of seven or more members. Adequate family support to the patient with T1D was reported by the majority (96%). A third of the patients were full-time employees, while 61% were students. Around 37.3% of the patients had a family history of T1D, and 47% reported a history of autoimmune disease in the family.

**Table 1:** Sociodemographic characteristics of Type 1 diabetes mellitus patients (n = 134).

	All n = 134 (%)	MDI n = 68 (50.7%)	CSII n = 66 (49.3%)	P-value
Age (mean ± SD)	20.96 ± 7.45	20.6 ± 8.1	21.3 ± 6.8	0.581
8–15 years	37 (27.6)	24 (35.3)	13 (19.7)	0.097
16–24 years	57 (42.5)	24 (35.3)	33 (50.0)	
25–40 years	40 (29.9)	20 (29.4)	20 (30.3)	
Gender				0.022
Male	58 (43.3)	36 (52.9)	22 (33.3)	
Female	76 (56.7)	32 (47.1)	44 (66.7)	
Mode of presentation for TID				0.762
DKA	62 (46.3)	30 (44.1)	32 (48.5)	
Symptomatic hyperglycemia	63 (47.0)	34 (50.0)	29 (43.9)	
None of the above *(accidentally discovered diabetes)*	9 (6.7)	4 (5.9)	5 (7.6)	
Duration of T1D				
<5 years	29 (21.6)	19 (27.9)	10 (15.2)	0.198
5–10 years	37 (27.6)	17 (25.0)	20 (30.3)	
>10 years	68 (50.7)	32 (47.1)	36 (54.5)	
Patients Education				0.021
Below high school (Grade 1–9)	30 (23.1)	21 (31.3)	9 (14.3)	
High school (Grade 10–12) and above	100 (76.9)	46 (68.7)	54 (85.7)	
Mother Education (for <18 years old patients)				0.891
Below high school	37 (30.6)	18 (30.0)	19 (31.1)	
High School and above	84 (69.4)	42 (70.0)	42 (68.9)	
Father’s education attainment (for <18 years old patients)				
Below high school	29 (24.0)	45 (73.8)	47 (78.3)	0.557
High School and above	92 (76.0)	16 (26.2)	13 (21.7)	
Family Size				0.619
<7 members	24 (19.2)	13 (21.0)	11 (17.5)	
≥7 members	101 (80.8)	49 (79.0)	52 (82.5)	
Family Support				
Adequate	119 (96.0)	61 (96.8)	58 (95.1)	0.622
Inadequate	5 (4.0)	2 (3.2)	3 (4.9)	
Patient occupation status				0.325
Unemployed	13 (9.8)	4 (6.0)	9 (13.6)	
Student	81 (60.9)	43 (64.2)	38 (57.6)	
Full time employee	39 (29.3)	20 (29.9)	19 (28.8)	
Family History of T1D				0.894
Yes	50 (37.3)	25 (36.8)	25 (37.9)	
No	84 (62.7)	43 (63.2)	41 (62.1)	
Family History of autoimmune diseases				0.389
Yes	62 (47.0)	29 (43.3)	33 (50.8)	
No	70 (53.0)	38 (56.7)	32 (49.2)	

MDI: Multiple daily insulin injections, CSII: Continuous subcutaneous insulin infusion.

The clinical characteristics of the included patients are depicted in Table 2. The mean body mass index (BMI) of T1D patients in this cohort was 25.4 ±4.8 kg/m^2^. The mean systolic and diastolic blood pressures (SBP & DBP) were 120.2 ± 10.3 and 73.3 ± 10.5 mmHg, respectively. As per the latest laboratory results, the overall median/IQR (mean ±SD) of the HbA1c, estimated glomerular filtration rate (eGFR), low density lipoprotein (LDL), high density lipoprotein (HDL), and triglycerides (TG) were 8.30/1.93% (8.6 ± 1.9%), 126.5/20.0 (119.9 ± 27.2 mL/min/1.73 m^2^), 2.4 ± 0.74 mmol/L, 1.47/0.63 (1.50 ± 0.50), and 0.76/0.70 (1.31 ± 2.8 mmol/L), respectively. Only four and five patients were on blood pressure lowering and lipid-lowering agents (statin), respectively. The overall median of vitamin D level was 48.5 mmol/L (IQR: 22) (mean: 50.0 ± 18.2 nmol/l in this cohort. Only 10.5% of the patients were actively smoking. Average Glycemic control (HbA1c) over the last 12 months was satisfactory, suboptimal, and poor in 26.6%, 42.7% & 30.6% of the patients, respectively. In this study, more than half (53.7%) of the patients reported self-monitoring blood glucose ≥ 4 times daily, and only 33.6% of the patients were using CGM/FGM at the study time. The recent biochemical investigations of the patients showed a lower (p = 0.041) median HbA1c % in patients using CSII (8.2/1.93%) compared to MDI (8.5/2.45%). There was no significant difference in eGFR, urine/albumin creatinine ratio, vitamin D level, and lipid profile between MDI and CSII group. We observe a statistically significant change in average glycemic control (>12 months) between the two groups. Higher proportions of patients using CSII therapy achieved suboptimal or satisfactory control compared to MDI (83.6% vs 55.6%).

**Table 2:** Clinical characteristics of Type 1 diabetes mellitus patients represented as Mean± SD, frequency or percentages.

	All n = 134 (valid %)	MDI n = 68 (valid %)	CSII n = 66 (valid %)	P-value
BMI (kg/m^2^)	25.4 ± 4.8	25.1 ± 4.5	25.8 ± 5.2	0.417
SBP (mmHg)	120.2 ± 10.3	119.2 ±10.8	121.3 ± 9.8	0.240
DBP (mmHg)	73.3 ± 10.5	71.9 ± 9.9	74.7 ± 10.9	0.129
Laboratory Findings				
HbA1c (median, IQR)	8.30, 1.93	8.50, 2.45	8.20, 1.93	(0.041)**
Males	8.35, 2.05	8.45, 2.23	8.35, 1.83	(0.718)**
Females	8.25, 2.03	8.55, 2.65	8.00, 1.98	(0.034)**
CGM/FGM not using	8.40, 2.20	8.30, 2.62	8.40, 2.00	(0.683)**
CGM/FGM using	8.10, 1.55	8.40, 1.98	7.90, 1.75	(0.047)**
eGFR (*mL/min/1*.*73m*^*2*^*)* (median, IQR)	126.5, 20.0	128.0, 24	126.0, 19	(0.985)**
Urine Alb/creatinine mean ratio (*mg/g)* (median, IQR)	0.70, 2.0	0.79, 2.0	0.63, 2.0	(0.759)**
LDL (*mmol/L)* (mean ± SD)	2.4 ± 0.74	2.5 ± 0.9	2.4 ± 0.6	0.912
HDL (*mmol/L)* (median, IQR)	1.47, 0.63	1.33, 0.68	1.51, 0.55	(0.137)**
Triglycerides (*mmol/L)* (median, IQR)	0.76, 0.70	0.88, 1.04	0.63, 0.48	(<0.001)**
Vitamin D (*mmol/L)* (median, IQR)	48.5, 22	46.5, 16	52.0, 25	(0.150)**
Current Smoker				0.332
Yes	10 (10.5)	6 (11.5)	4 (9.3)	
No	85 (89.5)	46 (88.5)	39 (90.7)	
Average glycemic control (HbA1C) over the last 12 months				0.003
Satisfactory	33 (26.6)	12 (19.0)	21 (34.4)	
Suboptimal	53 (42.7)	23 (36.5)	30 (49.2)	
Poor	38 (30.6)	28 (44.4)	10 (16.4)	
Satisfactory/Suboptimal	86 (69.4)	35 (55.6)	51 (83.6)	0.001
Daily frequency of blood glucose monitoring				0.847
Inconsistent	27 (20.1)	15 (22.1)	12 (18.2)	
≤3 times	35 (26.1)	17 (25.0)	18 (27.3)	
≥4 times	72 (53.7)	36 (52.9	36 (54.5)	
CGM or FGM use				0.014
Not using	75 (66.4)	46 (76.7)	29 (54.7)	
Using	38 (33.6)	14 (23.3)	24 (45.3)	

MDI: Multiple daily insulin injections, CSII: Continuous subcutaneous insulin infusion, BMI: Body Mass Index; SBP: Systolic Blood Pressure; DBP: Diastolic Blood Pressure; LDL-Low Density Lipoprotein; HDL: High Density Lipoprotein; CGM; Continuous Glucose Monitoring; FGM: Fasting Glucose Monitoring; DKA: Diabetes ketoacidosis, eGFR: Estimated glomerular filtration rate, LDL: Low density lipoprotein; HDL: High density lipoprotein.

** p-value obtained from comparing means of the non-normal distributed variables using the independent-samples Mann-Whitney U Test.

The annual frequency of visits to the physician, dietician, and diabetic educators is shown in Table 3. More than ninety percent of the patients in this cohort reported less than three annual visits to the dietician. Almost 45.5% of the patients reported DKA admission in the preceding 12 months, while 6% reported severe hypoglycemia episodes requiring assistance or hospitalization. Only five patients were diagnosed with celiac disease and 17 patients with thyroiditis or hypothyroidism.

**Table 3:** Follow up frequency and complications among Type 1 diabetes mellitus patients (n = 134).

	All n = 134 (%)	MDI n = 68 (%)	CSII n = 66 (%)	P-value
Number of annual visits to a physician				0.160
<3 times	61 (45.5)	35 (51.5)	26 (39.4)	
≥3 times	73 (54.4)	33 (48.5)	40 (60.6)	
Frequency seen by dietician over last 12m				0.714
<3 times	123 (91.8)	63 (92.6)	60 (90.9)	
≥3 times	11 (8.2)	5 (7.4)	6 (9.1)	
Frequency seen by diabetic educators over last 12m				
<3 times	40 (29.9)	31 (45.6)	9 (13.6)	<0.001
≥3 times	94 (70.1)	37 (54.4)	57 (86.4)	
Acute complications in the past 12 months				
DKA	61 (45.5)	29 (42.6)	32 (48.5)	0.498
Severe hypoglycemia *(with assistance or hospitalization)*	8 (6.0)	6 (8.8)	2 (3.1)	0.167
Long term complications (Retino-, Nephro-, Neuro-pathy, Hypertension, CHD, Cerebrovascular)				
No complications	80 (59.7)	38 (55.9)	42 (63.6)	0.549
Only one complication	43 (32.1)	23 (33.8)	20 (30.3)	
At least two complications	11 (8.2)	7 (10.3)	4 (6.1)	
Established any of these autoimmune Disease (*Celiac disease = 4*, *Thyroid disease = 17*, *Adrenal disorder & celiac = 1)*	22 (16.4)	8 (11.8)	14 (21.2)	0.140

Although we did not observe any statistically significant difference in daily frequency of blood glucose monitoring, the number of physician or dietician visits, but there was a statistically higher number of diabetic educators’ visits over 12 months’ period and a higher proportion of CGM/FGM use in CSII patients (45.3%) vs. MDI (23.3%). There was no statistically significant difference between the groups regarding episodes of DKA or hypoglycemia admissions over the preceding 12 months. Also, there was no significant difference in any long-term diabetes complications like retinopathy, nephropathy, neuropathy, hypertension, coronary heart disease (CHD) or cerebrovascular disease.

When WHO-5 Well-being Index applied to patients with T1D above the age of ≥18 years (n = 86), there was no statistically significant difference between CSII vs. MDI users in total scores or each item of the index except item WHO1 (I have felt cheerful in good spirits) (Table 4). In this regard, there was a statistical difference between CSII users MDI users (4.4 ± 0.9 vs. 3.6 ± 1.0, P = 0.014).

**Table 4:** Mean score of total and each item in WHO-5 well-being index as screening method for depression (n = 86 patients above the age of ≥18 years with T1DM).

The items the patient answered regarding how She/He felt in the last two weeks	MDI (n = 42) (Mean ± SD)	CSII (n = 44) (Mean ± SD)	P-value
WHO1: I have felt cheerful in good spirits.	3.6 ± 1.0	4.4 ± 0.9	0.014
WHO2: I have felt calm and relaxed.	3.6 ± 1.0	3.6 ± 1.2	0.990
WHO3: I have felt active and vigorous.	3.7 ± 1.0	3.4 ± 1.3	0.257
WHO4: I woke up feeling fresh and rested.	3.2 ± 1.2	3.3 ± 1.3	0.830
WHO5: My daily life has been filled with things that interest me.	3.9 ± 1.2	4.1 ± 0.96	0.542
Total	18.1 ± 3.8	18.1 ± 5.0	0.987

World Health Organization- Five Well-Being Index (WHO-5).

## 4. Discussion

The current cross-sectional study aimed to describe the sociodemographic and clinical characteristics of T1D patients in the Emirati population sample and compare those using CSII vs. MDI.

The frequency of DKA at the first presentation of T1D is variable between countries. In early systemic review, the frequency of DKA at diagnosis ranged from 12.8% to 80%, with the highest frequencies in the UAE, Saudi Arabia (SA), and Romania, and the lowest in Sweden, the Slovak Republic, and Canada [14]. With the increased level of disease awareness and healthcare provision, DKA frequency at presentation could decline, as has been observed in Finland, a country with the highest incidence of T1D [15].

In our cohort, almost half of the patients (46.3%) presented with DKA at the time of diagnosis, which is an excellent improvement from the previous study within UAE reported DKA in up to 80% of the patients at presentation [16]. Studies from regional countries like SA and Kuwait reported DKA in 45.25% (16) and 24.8% (6) at the time of diagnosis, while severe DKA was seen in 8.8% of patients [3]. In addition, high percentage (45.5%) included in the study had DKA episode in the last 12 months. Though, we did not explore the precipitating factors to DKA, giving the overall unsatisfactory glycemic control (median/IQR 8.30/1.93 (mean+ SD 8.6 ± 1.9%), and relatively overall young patients (age 20.96 ± 7.45 years) in this the cohort could be the reasons behind.

The National Institute for Health and Care Excellence (NICE) in the UK recommends target HbA1c of < 48 mmol/mol (6.5%) for both children and adults [17], while the most recent American Diabetes Association (ADA) guideline recommends a target < 53 mmol/mol (7%) for children and adults [18]. In our cohort, HbA1c over the last 12 months was satisfactory, suboptimal, and poor in 26.6%, 42.7% & 30.6% of the patients; consequently, with higher proportions of patients using CSII achieved suboptimal or satisfactory control compared to MDI. The overall median/IQR (mean ±SD) HbA1c was 8.30/1.93 (8.6 ±1.9%) as per the latest laboratory results, with a statistically lower median HbA1c in patients using CSII than MDI (8.2% vs. 8.5%). In comparison with various regional single-center studies in the Arabian Peninsula, this study’s findings are pretty coherent. E. Al-Agha *et al*. reported among children, HbA1c level of 8.8% with 70% of age group (5–10 years) with poor glycemic control (HbA1c >9) and 57.7% of the age group of post-puberty (>15 years) had poor control glycemic control [19]. Another single-center study from SA showed that among children and youth with T1D, the mean HbA1c was 9.6±1.9% and only 26.2% had satisfactory HbA1c (⩽8%) [20]. A study from Kuwait, comparing outcomes between CSII vs. MDI users, reported HbA1c of 8.9±1.4% and 8.8±1.4% in CSII and MDI patients, respectively at baseline [21]. Similar to our study’s findings, in a recent Canadian registry study, HbA1c was 8.1% ±1.5%, and only 22.5% had achieved HbA1c <7.0%. CSII was used by 39.3% of the patients, and showed a lower mean HbA1c than those using MDI [22].

Regarding CSII use over the MDI, most physicians consider CSII as a means of improving glucose control when MDI failed to reach and maintain desired therapeutic targets. Some guidelines, like the National Institute of Clinical Excellence (NICE) in the UK [23] and Diabetes Canada [24], recommend the switch to CSII when basal-bolus MDI does not provide satisfactory results, either for persistently elevated HbA1c or recurrent hypoglycemia. However, ADA guidelines stated that CSII might be considered an option for all adults and youth with T1D who can safely manage the device [25].

A higher proportion of T1D patients were on CSII (49.3%) in our cohort compared to other regional studies [20,21,26,27]. A selection bias in our study cannot be excluded as the cohort represents T1D subjects who agreed to participate in the initially designed study [28], and it involves only Emirates patients who have access to free healthcare service.

There is variability in the reported effectiveness of CSII over MDI in studies and practice. Some published meta-analyses of randomized controlled trials (RCTs) have indicated that the mean HbA1c difference between MDI and CSII is relatively small and not clinically significant, ~ 2.8 mmol/mol (0.25%) [7], or there is no statistically significant difference in the frequency of severe hypoglycemia between the two treatments in either adults or children with T1D [29]. On the other hand, in a multicentric population-based cohort study including patients with T1D<20 years of age, it has been shown that CSII therapy, compared with insulin injection therapy, was associated with lower risks of severe hypoglycemia and DKA and with better glycemic control during the most recent year of therapy [9]. Recent metanalysis showed that CSII produces a small improvement in HbA1c in patients with T1D inadequately controlled with MDI and that this improvement is smaller when MDI is correctly performed using a basal-bolus regimen with short-acting insulin analogs. In the same study, it has been reported no difference in severe hypoglycemia. Conversely, CSII was associated with a significant increase in the incidence of reported DKA, mainly in trials comparing CSII with conventional insulin therapy. In contrast, only a non-significant trend toward increased risk was observed compared with basal-bolus MDI [30].

In our cohort, higher proportions of patients using CSII therapy achieved suboptimal or satisfactory control than MDI, and on last lab investigations, median/IQR HbA1c % was lower in patients using CSII than MDI (8.2/ 1.93%vs. 8.5/ 2.45%). However, no statistical difference in terms of DKA or hypoglycemia admissions was observed in this study.

Overall, in this cohort of patients, it is obvious that the glycemic control in T1D patients in UAE is still not optimal. Though, patients using CSII have better overall glycemia control in this cohort, we need to emphasize that possibly patients who are uncontrolled on MDI are shifted to CSII which could count to the better glycemic control. On the other hand, the overall uptake of CGM is fairly low in patients with MDI compared to CSII which may not allow fair comparison between the two treatment modalities and possibly account for better control with CSII. In addition, more patients with education level of high school and above are encountered with CSII which could reflect a more independent patient who have a better self-control on CSII regimen. Though, we looked at the last HbA1c among MDI vs CSII users stratified by gender and CGM/FGM use, we do think it is of no clinical value. Stratification based on average HbA1c over the last 12 months (categorical variables) was difficult to look for with the small sample size and over stratification. Previous small local study showed that switching young patients with T1D from MDI regime to a specific type of CSII therapy with remote control achieved a reduction in HbA1c and insulin dose [31].

Regional data, including a study from Kuwait comparing CSII with MDI in children, showed that though HbA1c decreased most significantly in the first year, it continued to be significantly lower in the CSII group than the MDI throughout the study period. There was no significant change in the rate of DKA in either group. Although CSII patients had more severe hypoglycemic episodes at baseline, it significantly decreased throughout the study period [21].

T1D is associated with higher cardiovascular disease and death rates than the non-diabetic population, where the age of onset of T1D forming a critical determinant [32,33]. An observational study based on Swedish National Diabetes Register showed a lower adjusted hazard ratio for fatal coronary artery disease, fatal cardiovascular disease, and all-cause mortality among T1D patients using CSII than MDI. This was hypothesized to reduce numbers of severe hypoglycemia with CSII treatment [34]. Addressing cardiovascular risk factors; in our cohort, a minority of patients are actively smoking (10.5%), a mean BMI was 25.4±4.8, and acceptable mean BP control and lipid profile. However, almost 32.1% had one chronic diabetes-related complication. The higher latest Urine Alb/creatinine mean ratio (mg/g) observed in patients using CSII compared to MDI in this cohort could be due to the fact that these patients were in fact uncontrolled and then switched to CSII and it may be an imprecise estimate giving the fact that it reflects only one value (latest lab result). It is difficult to assess the adequacy of risk factors control with the current cross-sectional study. In addition, relatively young patients are included in our study.

An increased risk of autoimmune thyroid diseases (ATD) has been reported in patients with T1D with a wide range of estimated prevalence ranging from 17 to 30% [35–39]. This depends on the way looking for ATD, whether by screening with thyroid-stimulating hormone or thyroid autoantibodies. Also, it has been found that several factors such as age, female sex, duration of disease, and presence of beta-cell autoimmunity are associated with ATD in T1D patients [40]. Similarly, studies from the Gulf region showed a high prevalence of ATD among T1D, reaching up to a third of the patients having autoantibodies [38,39]. In our study, 17 out of 134 (10.4%) have ATD or hypothyroidism. This could be an imprecise estimation due to the study’s cross-sectional nature and the variability in screen for ATD in T1D patients among physicians.

In our cohort, the total WHO-5 raw score was 18.1, with overall no significant difference between CSII vs. MDI users. Giving the relatively young age of the subjects in our cohort, this may not reflect the true depression incidence or difference in patients with longer disease duration and across different complications severity.

The study has some notable strengths and limitations. Several studies reported on the comparison of CSII and MDI across the various population. However, this study is the first to our knowledge to compare the effectiveness of two insulin delivery regimens in T1D among the UAE populations. Apart from the small sample size, the inherent limitations of the cross-sectional and retrospective designs do apply to our study. There were missing data on some variables, including insulin doses. Furthermore, the two centers from where patients were collected are allocated in the same city with the same resources. This may not be generalizable to other parts of UAE. Future comprehensive multicentric, well designed, adequately powered, carefully controlled, cautiously conducted study would be advisable.

## 5. Conclusions

The present study characterizes the sociodemographic and clinical characteristics, metabolic control and well-being of T1D patients and compared the outcomes between CSII and MDI users.

In this cohort, the use of CSII is associated with better glycemic control than MDI, although no significant difference in admissions for DKA and hypoglycemia. Overall, the glycemic control among Emiratis subjects with T1D is suboptimal. Hence more rigorous patient counseling and education are needed for better compliance.

## Supporting information

S1 File (DOCX)
