# Supplementary material for: Continuous subcutaneous insulin infusion is associated with a better glycemic control than multiple daily insulin injections without difference in diabetic ketoacidosis and hypoglycemia admissions among Emiratis with Type 1 diabetes
Source: PLoS One. 2022 Sep 22;17(9):e0264545. doi: 10.1371/journal.pone.0264545 (PMC9498969; doi:10.1371/journal.pone.0264545)
Supplement: S1 File — (DOCX) [file pone.0264545.s001.docx]

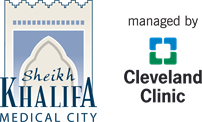


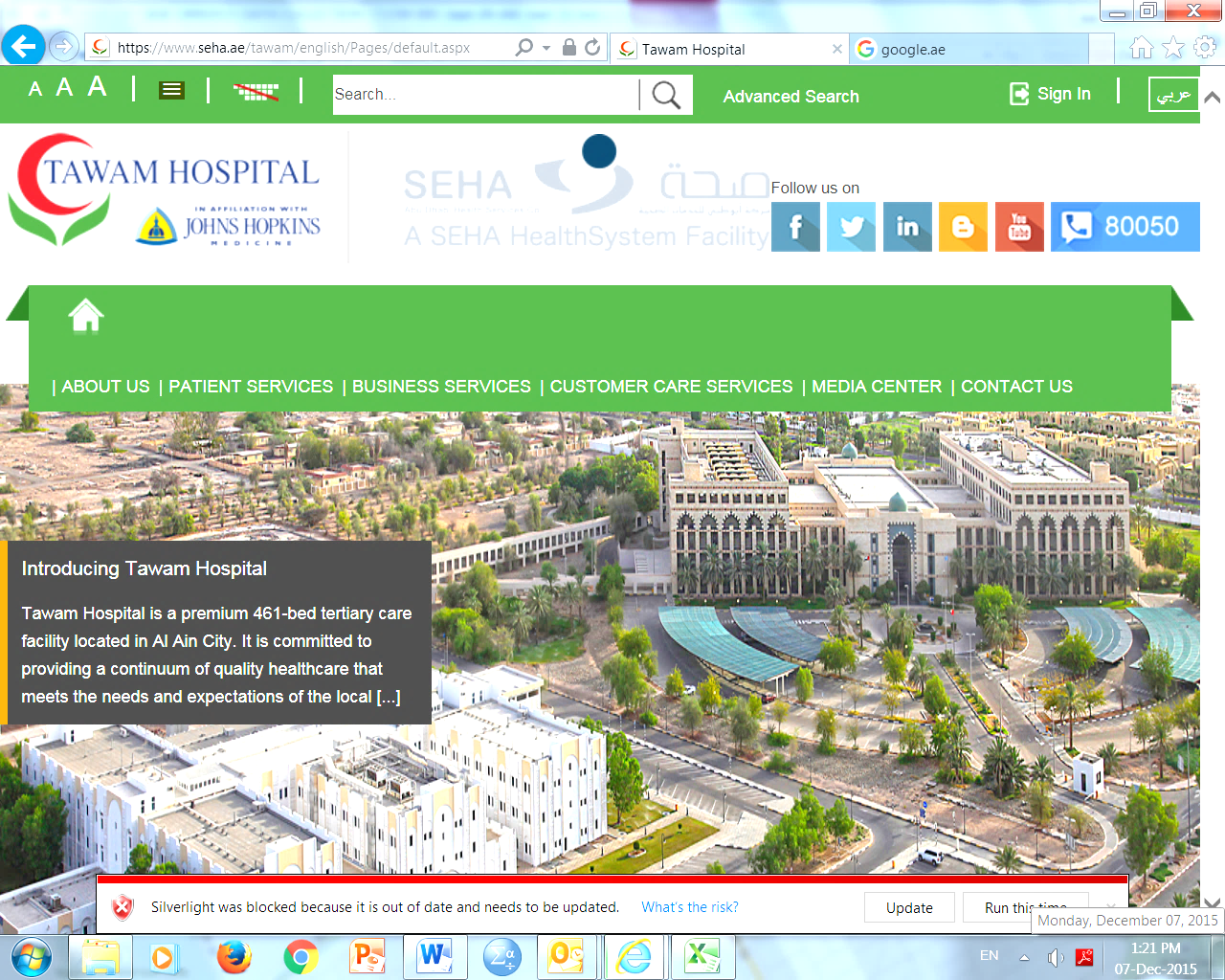

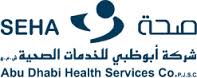

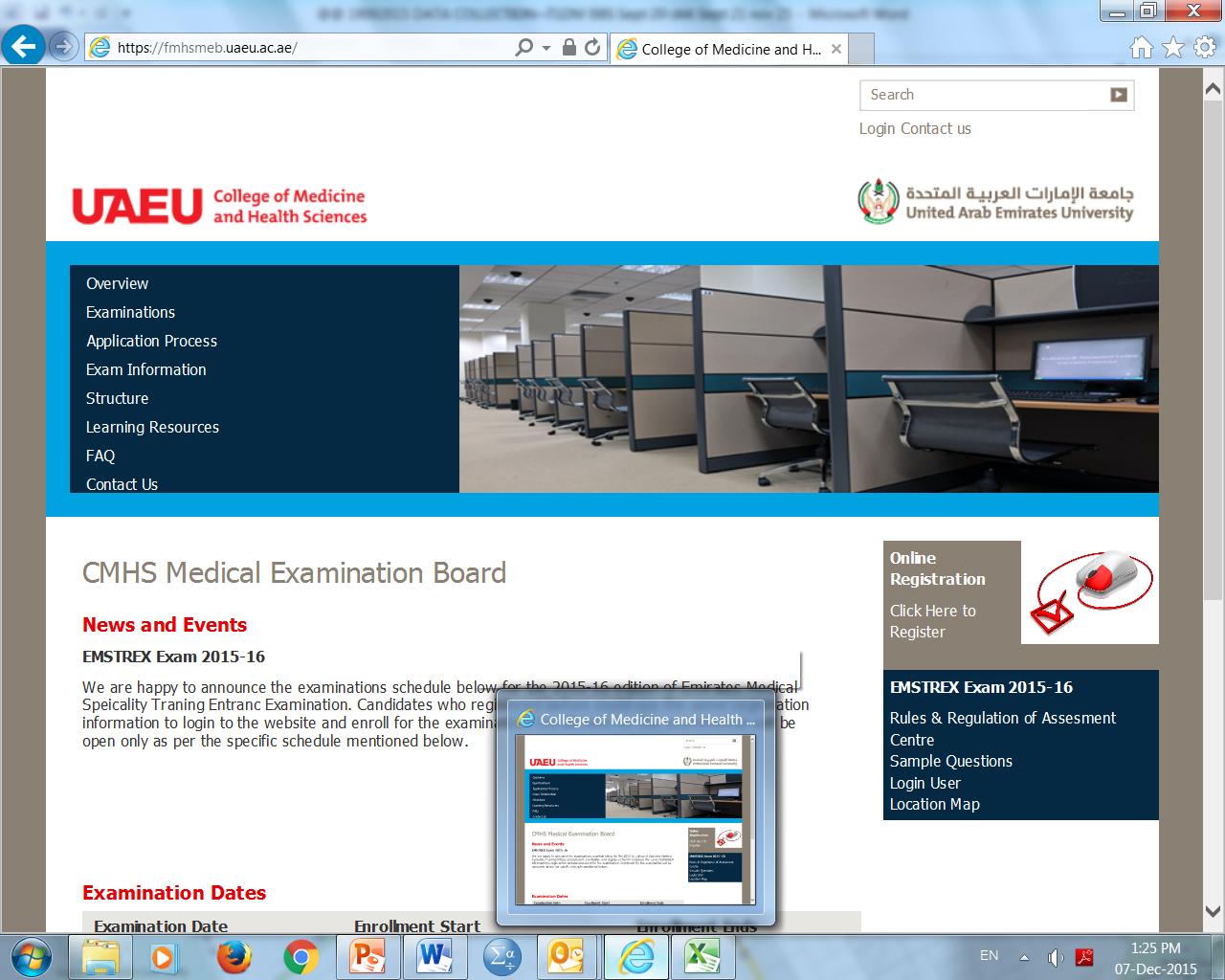

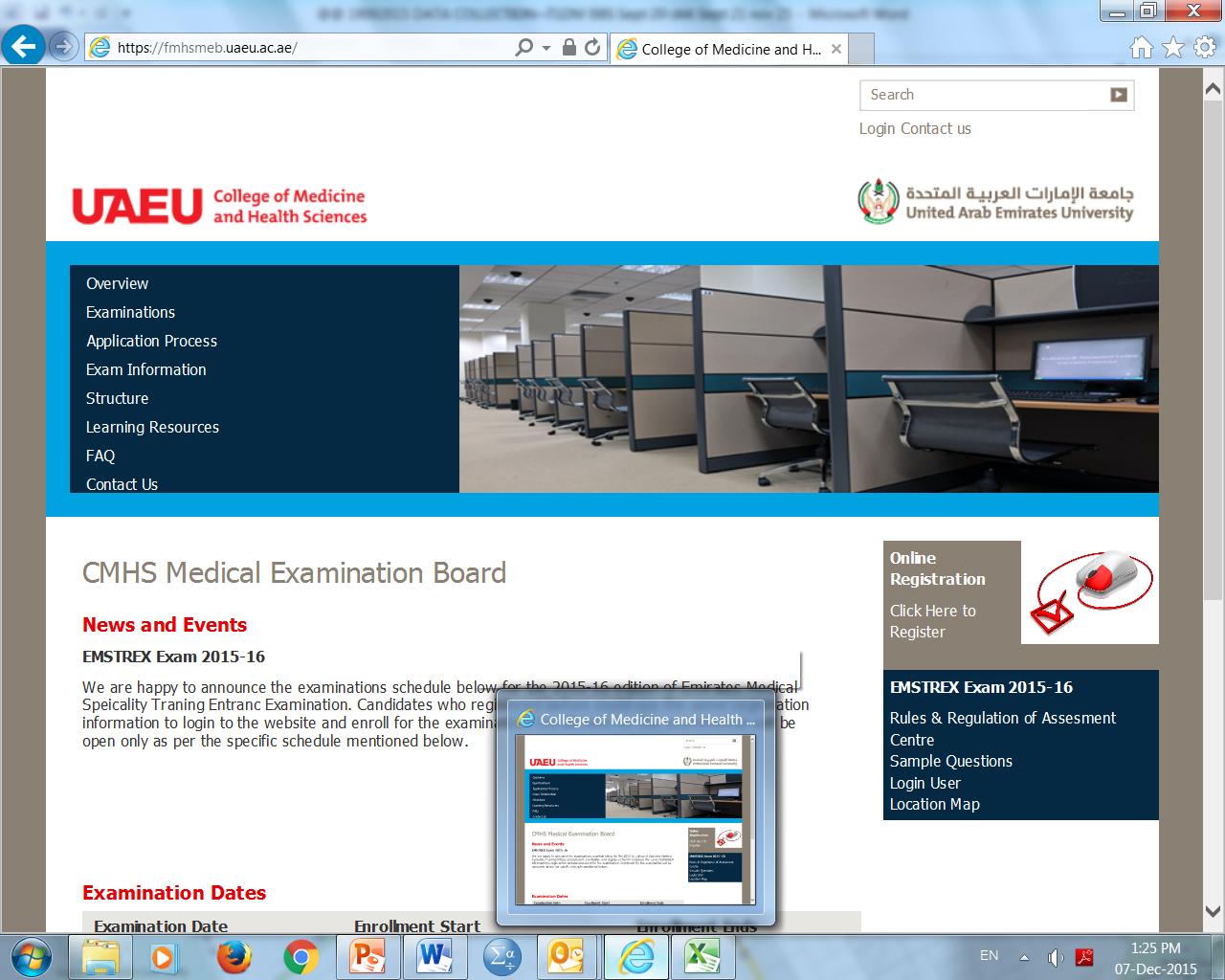


**Patients’ Data Collection Sheet**

**Glycemic control, Quality of Life and Genetic study of Emirati with Type 1 DM**

| **General Information** |
| --- |

- **Patient Serial Code**: _________________________________
- Geographical location: □ Al Ain □ SKMC
- DOB: ____/_________/ _____________________
- Gender: Male □ Female □
- Interviewer: _______________________________
- Date: ____________________________________

| **General info** | **Patient** | **Parents** | |
| --- | --- | --- | --- |
|  |  | **mother** | **father** |
| Parents Biologically Related | □ Non-related, □ cousin, □ distantly related | | |
| Birth delivery mode |  | X | X |
| Current Smoker |  |  |  |
| Secondhand smoking |  |  |  |

| **Education** | | |  |
| --- | --- | --- | --- |
| **Level** | **Patient** | **Mother** | **Father** |
| Grade 1-6 |  |  |  |
| Grade 7-9 |  |  |  |
| Grade 10-12 |  |  |  |
| University |  |  |  |
| Graduate |  |  |  |

| - Family size living in the same house:_______, Brothers ______, Sisters ______ - Family Support: □ adequate □ inadequate - Occupation: - □ Student - □ Working full time job - □ Working part time job - □ Not working |
| --- |

- Estimated date of diagnosis of DM (day/month/year):___/____/____________
- Mode of Initial Presentation: (you can choose more than one answer)
- □DKA
- □Symptomatic Hyperglycemia (Polyuria, Polydipsia, Weight loss)
- □Infection
- Others: specify:______________________________________________

- Confirmation of the diagnosis of DM based on the auto-antibodies: □Yes □No

- Average Glycemic control (HbA1C) over the last 12 months:

□Poor (More than 9%) □Suboptimal (7.5-9%) □Satisfactory (less than 7.5%)

- Frequency of visit to physician per year:
- □Less than 3
- □4
- □5
- □6
- □More than 6

- Average home daily frequency of blood glucose monitoring testing:
- □ Inconsistent
- □ 2
- □ 3
- □ 4
- □ More than 4

Is there current use of CGM or FGM? □ Yes □ No

- Seen by the Dietician over the last 12 months: □ none □ 1 □ 2 □ 3 □ 4 □ more than 4

- Seen by the Diabetic Educators over the last 12 months: □ none □ 1 □ 2 □ 3 □ 4

□ more than 4

Dietician and Diabetic educator’s reviews were counted from the chart

|  | |
| --- | --- |
| **Treatment** |  |

# Current Medications (Insulin and others if applicable):

| Names | Doses | Class |
| --- | --- | --- |
|  |  |  |
|  |  |  |
|  |  |  |

Approx. the total daily dose of insulin = …………….

Approx. The daily Basal Insulin = ……………..

Insulin Pump users:

Approx. daily basal/bolus ratio: …………………...

- Has treatment changed during the last 1 year? □ Yes □ No

| If yes, please describe: □ Type of Insulin □ Regimen ( MDI, Pump, TID,..) (you can choose more than one answer) |
| --- |
| **Co-Morbidities and Complications** |

- Complications:
- DKA in the past one year: □ Yes, □ No
- Hypoglycemia hospitalization in the past one year: □ Yes, □ No
- Major Failure to cope: □ Yes, □ No
- Stroke: □ Yes, □ No
- IHD: □ Yes, □ No
- Dental Problems: □ Nil, □Rarely, □ Sometimes, □ Common
- Have you ever had the following?

Retinopathy problem: □ Yes □ No Heart disease: □ Yes □ No

High blood pressure: □ Yes □ No Kidney disease: □ Yes □ No

Nerve disorders: □ Yes □ No Severe hypoglycemia □ Yes □ No

(numbness/tingling)

Cholesterol disturbances: □ Yes □ No

- Any other complication: □ Yes □ No

If yes for complications, please specify:___________________________________________

- Hospitalization with diabetes related problems: □ Yes □ No

If yes for hospitalization, please specify cause_____________________________________

- Previous surgery: □ Yes □ No

If yes, specify______________________________________________________________

- Established Autoimmune Diseases:
- Celiac disease □ Yes, □ No
- Thyroid □ Yes, □ No

If Yes specify:

□ Hypothyroidism □ Subclinical Hypothyroidism

□ Hyperthyroidism □Subclinical Hyperthyroidism

□ Goiter

- Adrenal disorders □ Yes, □ No
- Others: specify __________________________________________________________

| **Family history** |
| --- |

Family history: 1^st^ degree (siblings, mother, father) or 2^nd^ degree relatives (uncle, aunt, cousin, grandparent or half sibling) of any of the following:

- DM: □ Yes □ No

If Yes specify: □ Type 1, □ Type 2 □ others (you can choose more than one answer)

If yes for others, please specify ___________________________________________

- Autoimmune diseases: □ Yes □ No

If Yes specify: □ Thyroid □ celiac disease or □ others (you can choose more than one answer)

If yes for others, please specify ___________________________________________

- Hypertension: □ Yes □ No

**WHO) (Five) Well-Being Index**

WHO (Five) Well-Being Index will be utilized to assess the quality of life. This is validated questionnaire and available in the Arabic language.


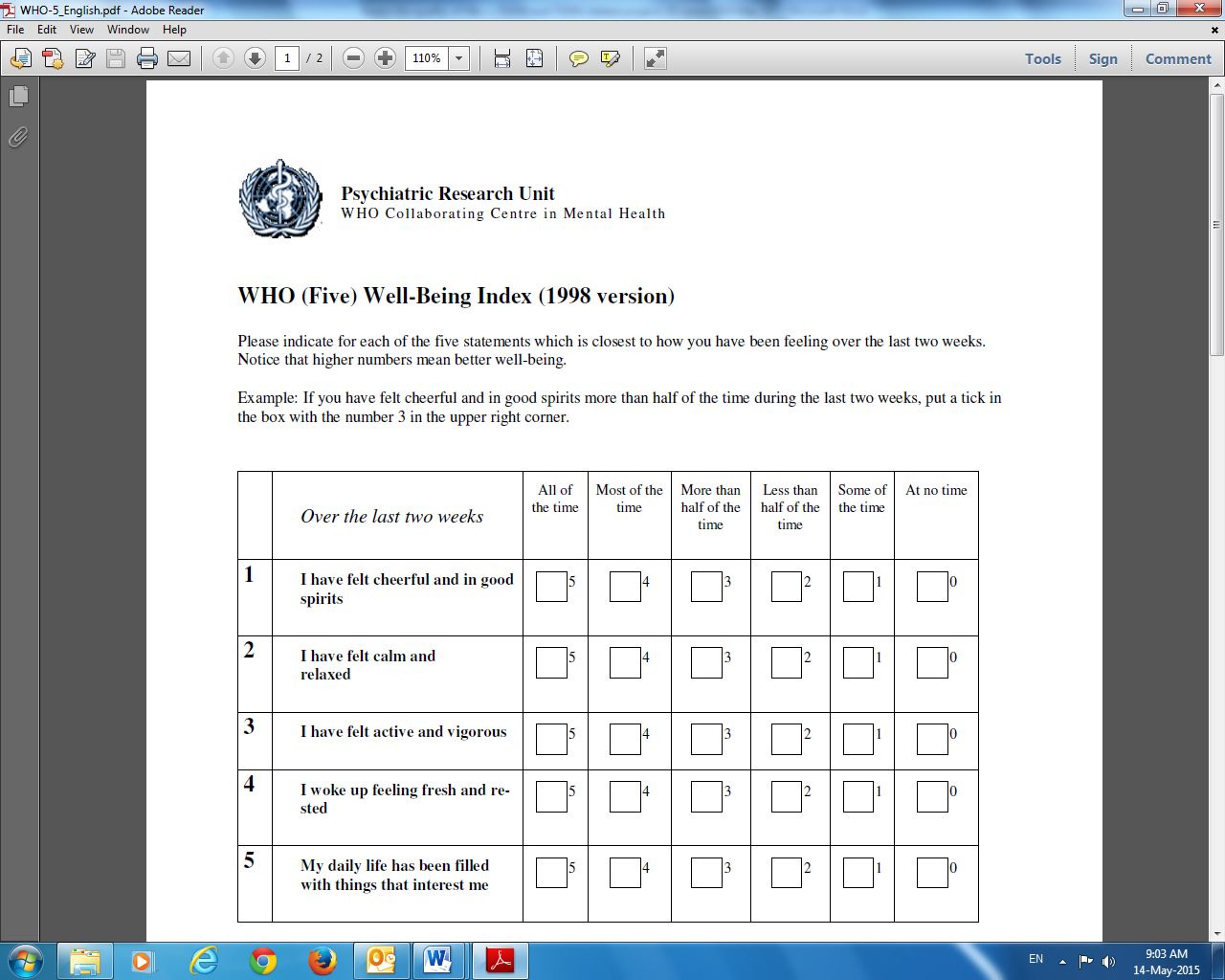


**Clinical assessment as per last clinic visit:**

Weight: ____________ Height:_________ BMI:___________ (children Percentile)

Systolic BP: ________ Diastolic BP:_________

Retinopathy: □ Present □ Absent □ NA

Neuropathy (10 grams filaments in 5 sites feet): □ Present □ Absent □ NA

Nephropathy: □ Present □ Absent □NA

**Investigations (most updated results):**

| Date | Parameter | Results |
| --- | --- | --- |
|  | HbA1c |  |
|  | eGFR |  |
|  | Urine Alb/creat ratio |  |
|  | LDL |  |
|  | HDL |  |
|  | TG |  |
|  | Vitamin D |  |
|  | IAAs |  |
|  | GAD65/ GADAs |  |
|  | IA-2As |  |
|  | Anti-TPO |  |
|  |  |  |
|  |  |  |
|  |  |  |

Note: Anti-bodies results is available only.
